# Paternal Exposure to Methylphenidate Induces Poor‐Quality Blastocyst and Epigenetic Changes

**DOI:** 10.1002/mrd.70026

**Published:** 2025-05-23

**Authors:** Ana Clara da Costa Nunes Gomes, Laura Eduarda S. C. Pagliari, Taiza Stumpp, Vanessa Vendramini

**Affiliations:** ^1^ Department of Morphology and Genetics, Laboratory of Reproductive and Developmental Biology (LaBReD) Paulista School of Medicine Federal University of Sao Paulo ‐ EPM/UNIFESP São Paulo Brazil

**Keywords:** embryo, histone methylation, psychostimulant, spermatozoa, toxicology

## Abstract

Epigenetic changes caused by methylphenidate hydrochloride on paternal inheritance have been suggested in fish, yet a subject to be determined in mammals. In rats, we showed increased sperm DNA fragmentation and reduced embryonic viability. In the present report, male *Wistar* rats (*n* = 21) were divided into two groups: control and methylphenidate. The control group received 1 mL/kg of distilled water, while the methylphenidate group received 5 mg/kg by gavage from 38 to 68 days of age on a single daily dose. After this period, there was an interval before exposed rats started a mating schedule with untreated/normally cycling females. Morphological quality and key epigenetic marks in the blastocysts were assessed. Immunocytochemistry was performed in fresh blastocysts to quantify the trimethylated histones H3K4, H3K9, and H4K20. Treatment with methylphenidate reduced the mean quality of blastocysts by 43.57% (*p* = 0.02), as well as increased those classified as “poor” by more than 150% (*p* < 0.001). Epigenetic marks were also altered, with an increase in the intensity of H3K9me3 (*p* = 0.01), a reduction of H4K20me3 (*p* = 0.05) and a nonsignificant increase of H3K4me3 (*p* = 0.34). The results suggest that the decline in blastocyst quality is highly associated with subchronic use of this psychostimulant by adolescent males. This is the first report showing the risks posed by methylphenidate to the epigenetic signature of a mammalian blastocyst following paternal exposure.

**Abbreviations**
ADHD: attention deficit hyperactivity disorder
ATM: ataxia telangiectasia mutant protein kinase
CNS: central nervous system
DA: dopamine
DFI: DNA fragmentation index
DSBs: double‐strand breaks
HP1: heterochromatin protein 1
MPH: methylphenidate hydrochloride
MPH group: methylphenidate‐treated rats
NE: noradrenaline
PBS: phosphate‐buffered saline
PRMs: protamines
PTMs: posttranslational modifications
SART: Society for Assisted Reproductive Technology
SC group: sham control rats
TNPs: nuclear transition proteins

## 1 Introduction

For more than half a century, methylphenidate hydrochloride (MPH) has been the major pharmacological therapy for attention deficit hyperactivity disorder (Storebø et al. 2018), representing a risk of developing substance abuse (Leahy 2017). Long‐term implications on male reproductive health following exposure deserve more attention, considering that dopamine receptors are expressed in spermatogonia (C. R. González et al. 2015).

In humans, few reports suggest that it can cause alterations in conventional seminal parameters, including a reduction in motile sperm count and seminal volume without affecting sperm morphology (Pham et al. 2022; Shalev et al. 2021; Aliakbari et al. 2022). In animal models, broader effects were observed in the testicular tissue (Fazelipour et al. 2012; Fazelipour et al. 2014) and spermatogenesis (Cansu et al. 2011; Kianifard et al. 2013; Montagnini et al. 2014).

We were the first to report genotoxic effects in mammalian sperm and negative implications to embryonic development caused by exposure during adolescence (da Costa Nunes Gomes et al. 2022). Moreover, evidence of intergenerational transmission through paternal exposure have been shown in fish (De Serrano et al. 2021), and more recently in mouse (Nakano et al. 2023).

Sperms are endowed with a very specific epigenetic profile that is essential for preimplantation embryonic development, also susceptible to modifications in response to exposure to xenobiotics (Cescon et al. 2020; Terrazas‐Salgado et al. 2022). Faulty DNA methylation landscape (Skinner et al. 2018), retained histones with inappropriate posttranslational modifications (PTMs) (Hammoud et al. 2015; Pandya et al. 2024), and altered/missing cargo of ncRNAs acquired during spermatozoa transit through the epididymis (Conine et al. 2018) are related to poor reproductive outcomes. Noteworthy, altered amounts of histones that remain attached to sperm DNA are related to increased sperm DNA fragmentation (Yoshida et al. 2018), predisposing the offspring to carry epigenetic errors. In male germ cells, the methylation profile of histones H3K4, H3K9, H3K27, and H4K20 have a particular strong epigenetic impact (Chioccarelli et al. 2020; Barbero et al. 2023). Concerning the paternal inheritance, as sperm chromatin is not completely epigenetically reprogrammed after fertilization, undue changes in histone PTMs can be transmitted to the preimplantation embryo (Lismer and Kimmins 2023).

In the present study, we point out that the high levels of DNA fragmentation caused by the subchronic treatment with MPH with a moderate dosage, previously reported by our group, are directly associated with morphological alterations of blastocysts and disturbance to their epigenetic identity.

## 2 Methods

### 2.1 Animals

Twenty‐one male and 48 female *Wistar* rats (*Rattus norvegicus* alb.) aged 4 and 8 weeks, respectively, were obtained from the Center of the Development of Experimental Models for Medicine and Biology (CEDEME/UNIFESP, Sao Paulo, Brazil). They were then kept at the Laboratory of Reproductive and Developmental Biology (LaBReD/UNIFESP) for a 7‐day adaptation period before the experiment began. The rats were housed in polypropylene cages, with up to four animals per cage, under standard controlled conditions, including hygiene, temperature (22°C–25°C), humidity (45%–60%), and a 12‐h light/dark cycle. The rats were provided with food (Labina, Purina, Paulínia, Brazil) and water ad libitum.

### 2.2 Ethical Approval Statement

The Ethics Committee on the Use of Animals of the Federal University of Sao Paulo (CEUA/UNIFESP) approved the experimental protocol, which adhered to the ethical principles established by the Brazilian School of Animal Experimentation. The protocol was assigned the number 2894060421.

### 2.3 Experimental Design

**Figure 1:**
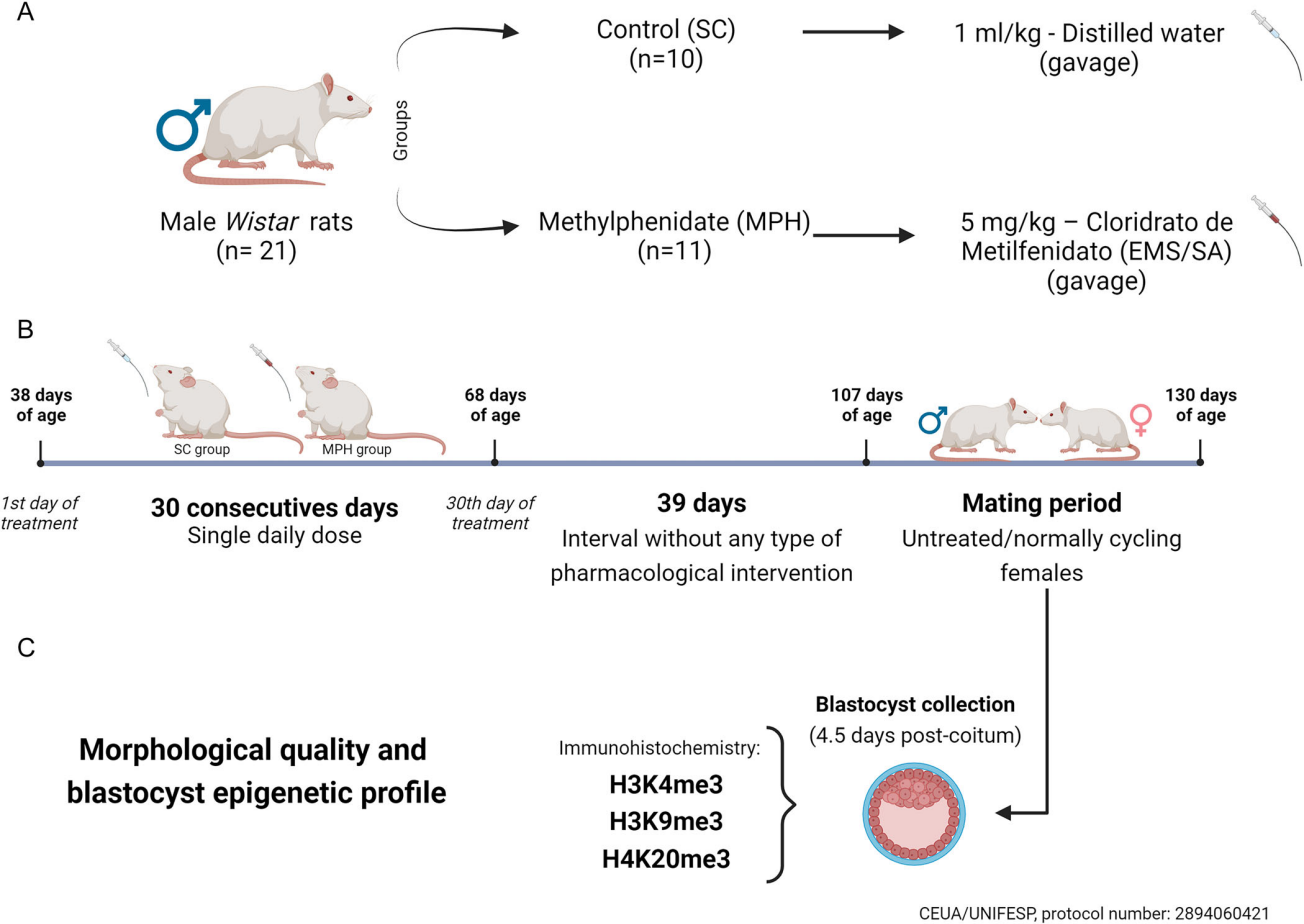
Experimental design. (A) Treatment protocol: male *Wistar* rats (*n* = 21) were divided into two groups: sham control (SC) and methylphenidate hydrochloride (MPH) (*n* = 10–11/group). The SC group received 1 mL/kg of distilled water, while the MPH group received 5 mg/kg of methylphenidate (Cloridrato de metilfenidato, EMS/SA) orally by gavage. (B) Treatment schedule: the animals received their respective treatments, from 38 to 68 days of age, in a single daily dose, totaling 30 consecutive days of administration. After this period, there was an interval of 39 days without any intervention until 107 days of age, when these animals were mated with untreated/normally cycling females. (C) Morphological quality and epigenetic profile of blastocysts: at 4.5 days postcoitum, blastocysts were collected, morphologically evaluated, and subjected to immunohistochemistry to quantify the fluorescence intensity of the epigenetic marks: H3K4me3, H3K9me3, and H4K20me3. Panel created with BioRender.com.

At 38 days old, male rats were randomly distributed into two groups: sham control (SC; *n* = 10) and methylphenidate group (MPH; *n* = 11). The SC group received only distilled water (1 mL/kg of body weight), while the MPH group received a single daily dose of 5 mg/kg body weight of MPH (Cloridrato de metilfenidato, EMS/SA, Sao Paulo, Brazil) diluted in distilled water for 30 days by gavage (Figure 1A).

Most preclinical studies on MPH were performed in rodents by injection (intraperitoneal, intravenous, or subcutaneous), differing from oral administration in the clinical practice. Since the route of administration is directly related to the magnitude and time of serum concentration, half‐life, and elimination rate, we chose gavage as administration route applying the BW3/4 scale. Moreover, to approach equivalence to clinically relevant dose in periadolescents (0.3–1 mg/kg), we applied the allometric scaling (Nair and Jacob 2016; Montagnini et al. 2014).

At the end of the treatment period, and after 39 days without any pharmacological intervention, male rats from both groups, at 107 days of age, were mated until 130 days of age (a time period necessary to contemplate all animals) with healthy and untreated/normally cycling females (*n* = 48) to evaluate the morphological quality and epigenetic profile of blastocysts (4.5 days post coitum) as presented in (Figure 1B). Each male rat was mated overnight with females in the proestrus phase. At the following morning (day 1 of gestation), female rats were checked for pregnancy; the test was considered positive when spermatozoa were found in vaginal smears (Vendramini et al. 2012).

Throughout the study, the rats were assessed daily for body weight before treatment, and their health was evaluated periodically by a veterinarian for any indications of symptoms, such as lacrimation, piloerection, abnormal respiratory patterns, tremors, nosebleeds, and tongue swelling. Figure 1 illustrates the complete experimental design.

### 2.4 Euthanasia and Collection of Samples

On the morning of the fifth day after mating (4.5 dpc), female rats were anesthetized by intraperitoneal injection of Dopalen/Anasedan solution containing ketamine (100 mg/kg of body weight, or 1 mL/kg of Dopalen) and xylazine hydrochloride (13 mg/kg of body weight, or 0.6 mL/kg of Anasedan). Laparotomy was performed followed by diaphragm rupture and chest decompression (Vendramini et al. 2012). The female reproductive organs were collected (ovaries and uterine horns), trimmed free of fat in a Petri dish, and placed under a stereomicroscope (M125, LEICA) for isolation of the ovarian capsule and revelation of the infundibulum. Using tweezers and a syringe with a 26G needle inserted into the infundibulum ostium, the oviducts and uterine horns were flushed with 1 mL of phosphate‐buffered saline (PBS) (pH = 7.4) previously heated (37°C). This procedure was repeated three times, ensuring the release of embryos.

### 2.5 Morphological Quality of Blastocysts

Initially, the collected embryos were categorized into morulas, blastocysts, and degenerates. Blastocysts were classified morphologically according to the parameters defined by Gardner et al. (2000), which takes into account: blastocoel expansion, inner cell mass (ICM), and trophoectoderm. Furthermore, the criteria applied by the Society for Assisted Reproductive Technology (*SART*) were also considered, grading them as good, fair, or poor (Heitmann et al. 2013). On the basis of these results, using the model proposed by Moriyama et al. (2022), scores were assigned to the categorical variables determined by the criteria mentioned above, transforming them into numerical variables. In this way, it was possible to apply the statistical analysis of the “Quality Index,” carried out by multiplying the scores obtained from the criteria of Gardner et al. (2000), as exemplified in Table 1. To visualize the embryos, the AZ100 Multizoom microscope (Nikon, Sao Paulo, Brazil) provided with phase contrast under magnification ranging from ×15 to ×40 was used.

**Table 1:** Scoring criteria used for the morphological classification of the blastocysts. Adapted from Moriyama et al. (2022).

Gardner et al. (2000)	
*Blastocoel*	
1. Cavitation occupying less than half the volume of the embryo—*SCORE1	
2. Blastocoel occupying more than half the volume of the embryo—*SCORE2	
3. Blastocoel occupying the whole volume of the embryo—*SCORE3	
4. Expanded blastocyst and thin zona pellucida—*SCORE4	
5. Hatching blastocyst—*SCORE5	
6. Hatched blastocyst—*SCORE6	
*Internal cell mass (ICM)*	
A. Many cells—*SCOR3	
B. Some loose cells —*SCORE2	
C. Few large cells—*SCORE1	
*Trophoectoderm (TE)*	
A. Many cells and cohesive epithelium—*SCORE3	
B. Few cells and loose epithelium—*SCORE2	
C. Very few large cells—*SCORE1	

SART	Quality Index—Moriyama et al. (2022)
*GOOD*	*FORMULA USING GARDNER AND SART*
AA or AB—*SCORE3	*Blastocoel* × *ICM* × *TE*
*REGULAR*	
BB, BC, or BA—*SCORE2	*For example, 3BC for Gardner/regular for SART*
*BAD*	QUALITY INDEX = 3 × 2 × 1 = 6
CC or CB—*SCORE1	

Abbreviation: MPH, methylphenidate hydrochloride.

### 2.6 Immunocytochemistry

To check the epigenetic profile of the blastocysts, only those displaying the typical morphology expected for this stage of development—containing well‐defined ICM, trophectoderm, and blastocoel—were selected to the immunocytochemical study, for the analyses of histones H3K4me3, H3K9me3, and H4K20me3. The steps for immunostaining were carried out according to the protocol described by Barton et al. (2005), with some modifications. Embryos were collected in PBS (pH = 7.4), then washed in 1% polyvinylpyrrolidone (PVP/PBS) and fixed in 4% paraformaldehyde (PFA/PBS) for 15 min.

Afterward, they were washed in 0.05% Tween‐20/PBS, permeabilized with 0.2% Triton X‐100/PBS for 20 min, and washed again in 0.2% Tween‐20. Subsequently, they were blocked in 1% bovine serum albumin (BSA) solution for 2 h at room temperature and then incubated overnight at 4°C with the primary antibodies of interest: anti‐H3K4me3 (1:200; ab8580, Abcam), anti‐H3K9me3 (1:500; ab8898, Abcam), and anti‐H4K20me3 (1:500; ab177190, Abcam) diluted in PBS. Furthermore, negative controls were performed, where some blastocysts were not exposed to the incubation step with primary antibodies. In the morning of the following day, after three washes in 1% BSA for 20 min each, the blastocysts were incubated with the secondary antibody Alexa Fluor 488 (1:500; ab150077, Abcam) diluted in PBS for 1 h at room temperature.

Finally, they were washed twice in 1% BSA (20 min each) and then stained with 4′,6′‐diamino‐2‐phenyl‐indole (1:1000; ab285390, Abcam) to the identification of nuclei, for 20 min. The blastocysts were then mounted on a slide with glycerol, which was covered with a coverslip and sealed with toluene sulfonamide/formaldehyde resin.

The samples were analyzed using a confocal microscope equipped with a multiphoton system (Leica TCS SP8, Mannheim, Germany) under a 40× objective. Using the single‐plane image stacking mode, known as “*z*‐stack” a series of images were acquired from horizontal planes, sized 1024 × 1024, pixel depth of 8 bits, and maintaining the pattern of 27 layers or “sections” per blastocyst, so that the first and last planes were positioned at the limits of the embryonic structure. The software used to analyze the images was ImageJ (1.53t; National Institutes of Health), which processed and analyzed the projections obtained in *Z* (*z*‐stack). The sum projection (SUM project) was used, which adds all pixels with the same *xy*‐coordinates, each pixel value influences the result, so that the fluorescence intensities in the projection image were evaluated appropriately.

To quantify the total signal intensity of each blastocyst, the fluorescence area in each nucleus was selected, and the intensity was calculated after removing the automatic nonspecific background. The background was also removed manually to apply the corrected total cell fluorescence formula (CTCF = Integrated Density − [Area of selected cell × Mean fluorescence of background readings]), allowing greater reliability of the results (Fitzpatrick 2021). The intensity of total fluorescence was expressed in arbitrary units.

### 2.7 Statistical Analysis

All data from the present study were submitted to normality (Shapiro–Wilk) and homogeneity (Levene) tests. The Student's *t* test, with Welch's correction for data with unequal variances, was performed to identify statistical differences between groups SC and MPH, considered significant when *p* ≤ 0.05. The results were obtained using the Jamovi software (version 2.2.5), and the graphs were generated using GraphPad Prism (version 8.0).

## 3 Results

### 3.1 Morphological Analysis

**Figure 2:**
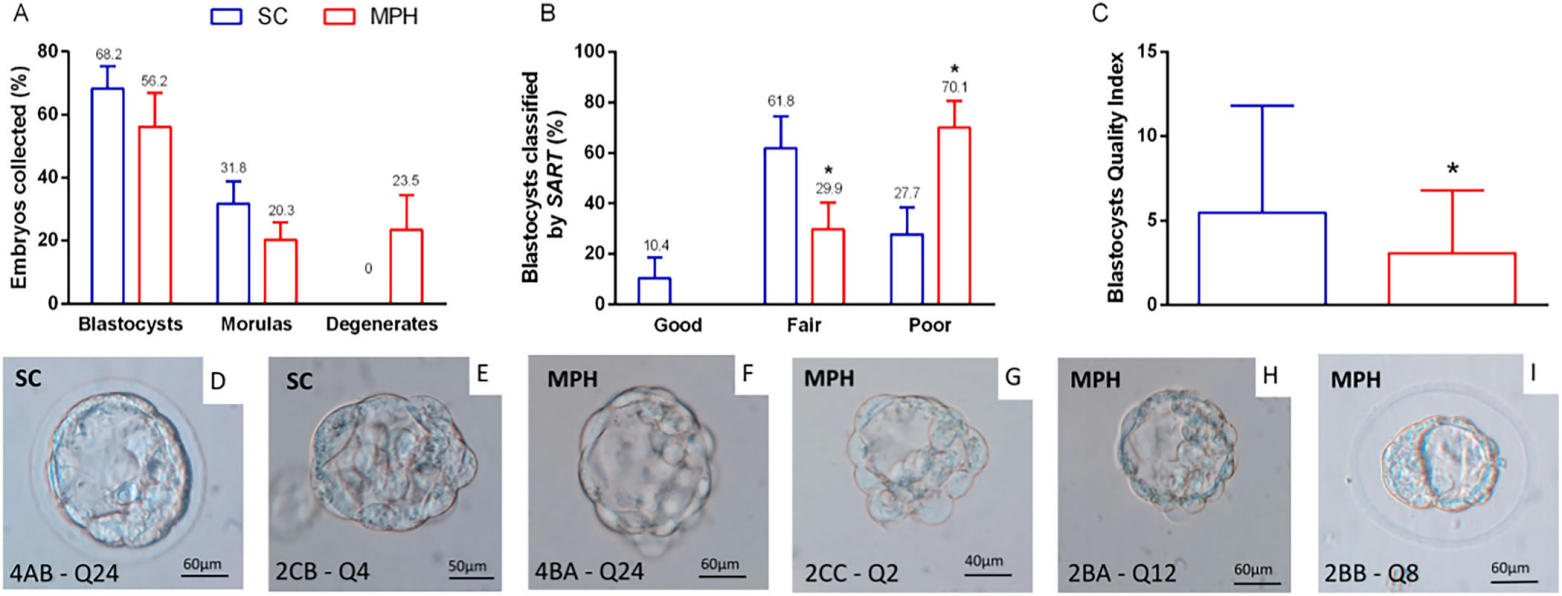
Morphological analysis at blastocyst stage. (A) Embryos (*n* = 171) were collected for morphological analysis, identified as blastocysts, morulas, and degenerates. The relative number of embryos among categories was not statistically different when comparing groups SC and MPH (blastocyst, *p* = 0.41; morulas, *p* = 0.22; degenerates, *p* = 0.06). Statistical analysis was performed using the Student's *t* test, with Welch's correction for “morulas,” and “degenerates” data (*n*/group, SC = 68 and MPH = 103). (B) Using the SART criteria, blastocysts (*n* = 107) were morphologically classified as “good,” “fair,” or “poor.” In the MPH group, no “good” embryos were identified, and a statistically higher number of blastocysts were classified as “poor,” and a reduced number was classified as “fair” when compared with those from group SC. The groups were compared using the Chi‐square test (*p < 0.001; *n*/group, SC = 47 and MPH = 60). (C) The blastocyst quality index, proposed by Moriyama (2022), refers to the criteria established by Gardner et al. (2000). Blastocysts from the MPH group showed a statistically significant reduction in quality when compared with those from group SC. Student's *t* test, with Welch's correction, was applied in this analysis (*p = 0.02; *n* per group, SC = 47 and MPH = 60). All the values represent means and standard errors. (D–I) Examples of the morphological characteristics of the blastocysts; in (D) and (E), blastocysts are classified as good (4AB—Q24) and poor (2CB—Q4), respectively. In (F)–(I), the blastocysts of the MPH group presented more frequently embryoblast and trophectoderm composed of fewer cells disposed in a less organized manner, which were classified as (G, H), but fair blastocysts, considered resilient, were also seen (2BB—Q8, in I). MPH, methylphenidate hydrochloride; SART, Society for Assisted Reproductive Technology; SC, sham control.

In the morphological analysis, 171 embryos were analyzed, being 68 descendants of SC animals and 103 of MPH animals. The embryos were identified according to the presented features and categorized as blastocysts, morulas, or degenerates. Representative images of blastocysts are presented in Figure 2D‐I.

Proportionally, no statistically significant differences were observed in embryo morphology between the groups, despite the MPH group presenting a notable percentage of degenerated embryos (Figure 2A). When we applied the criteria defined by *SART* (Heitmann et al. 2013) (Figure 2B,D–I), considering only the morphology of the blastocysts (*n* = 107; SC = 47 and MPH = 60), the majority (70.1%) of those conceived by males of the MPH group were classified as “poor” surpassing the scores of the males from SC by more than two times (27.7%). Furthermore, no embryo of the MPH group was classified as “good” and embryos classified as “fair” were considerably reduced (61.8% SC vs. 29.9% MPH).

According to the quality index, embryos conceived by MPH‐treated males were scored as “low quality” with a statistically significant difference when compared with group SC (Figure 2C–I).

### 3.2 Histone Methylation at the Blastocyst Stage

**Figure 3:**
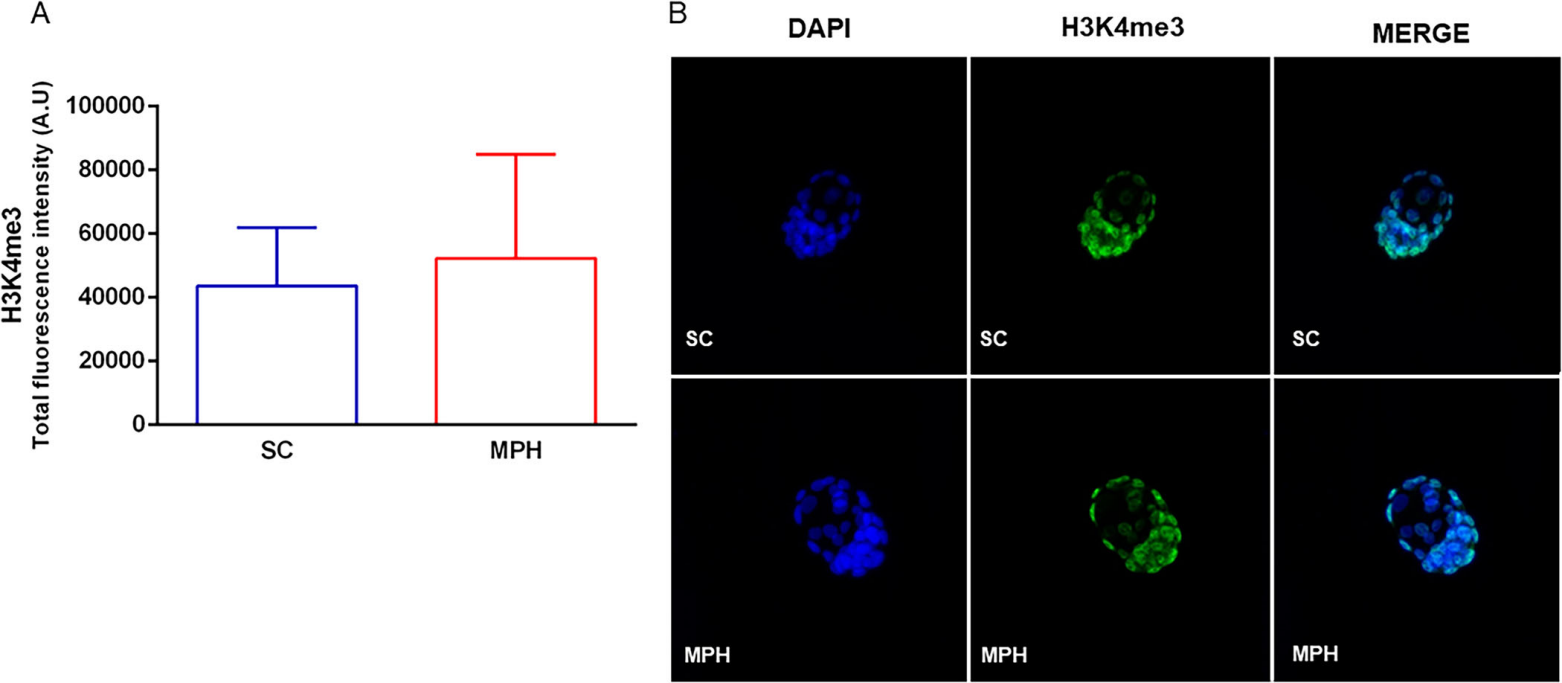
Epigenetic profile of H3K4me3 in blastocysts by immunofluorescence. (A) Quantification of total fluorescence intensity for H3K4me3 did not differ between SC and MPH groups (*p* = 0.34; *n* of blastocysts/3–4 males/group, *n* = 16 [SC] and *n* = 18 [MPH]). Statistical analysis was performed using Student's *t* test, with Welch's correction. (B) Representative image of immunocytochemical staining for H3K4me3 in blastocysts. Nuclei were stained with DAPI (blue) and H3K4me3 with Alexa488 (green). DAPI, 4′,6′‐diamino‐2‐phenyl‐indole; MPH, methylphenidate hydrochloride; SC, sham control.

A total of 135 blastocysts were analyzed for tri‐methylation of histones H3K4, H3K9, and H4K20, quantified from the total fluorescence intensity generated by immunocytochemical assay. For H3K4me3, classically associated with gene activation, no significant differences were observed when comparing the fluorescence intensities of group SC with the MPH group (Figure 3A,B; *n* = 16 SC; *n* = 18 MPH).

**Figure 4:**
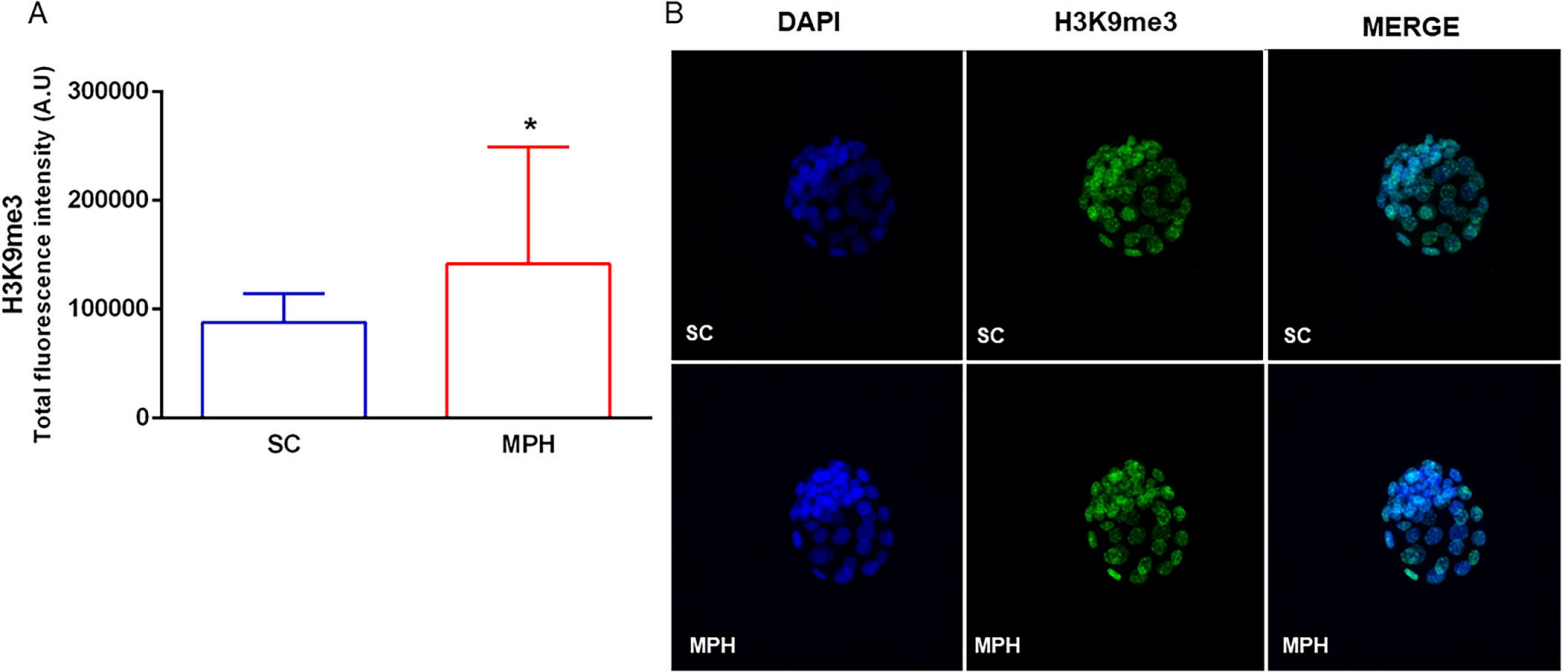
Epigenetic profile of H3K9me3 in blastocysts by immunofluorescence. (A) Blastocysts from the MPH group showed a statistically significant increase in total fluorescence intensity for H3K9me3 when compared with the SC group (*p = 0.01; *n* of blastocysts/4 males/group, *n* = 20 [SC] and *n* = 32 [MPH]). Statistical analysis was performed using Student's *t* test, with Welch's correction. (B) Representative image of immunocytochemical staining for H3K9me3 in blastocysts. Nuclei were stained with DAPI (blue) and H3K9me3 with Alexa488 (green). DAPI, 4′,6′‐diamino‐2‐phenyl‐indole; MPH, methylphenidate hydrochloride; SC, sham control.

**Figure 5:**
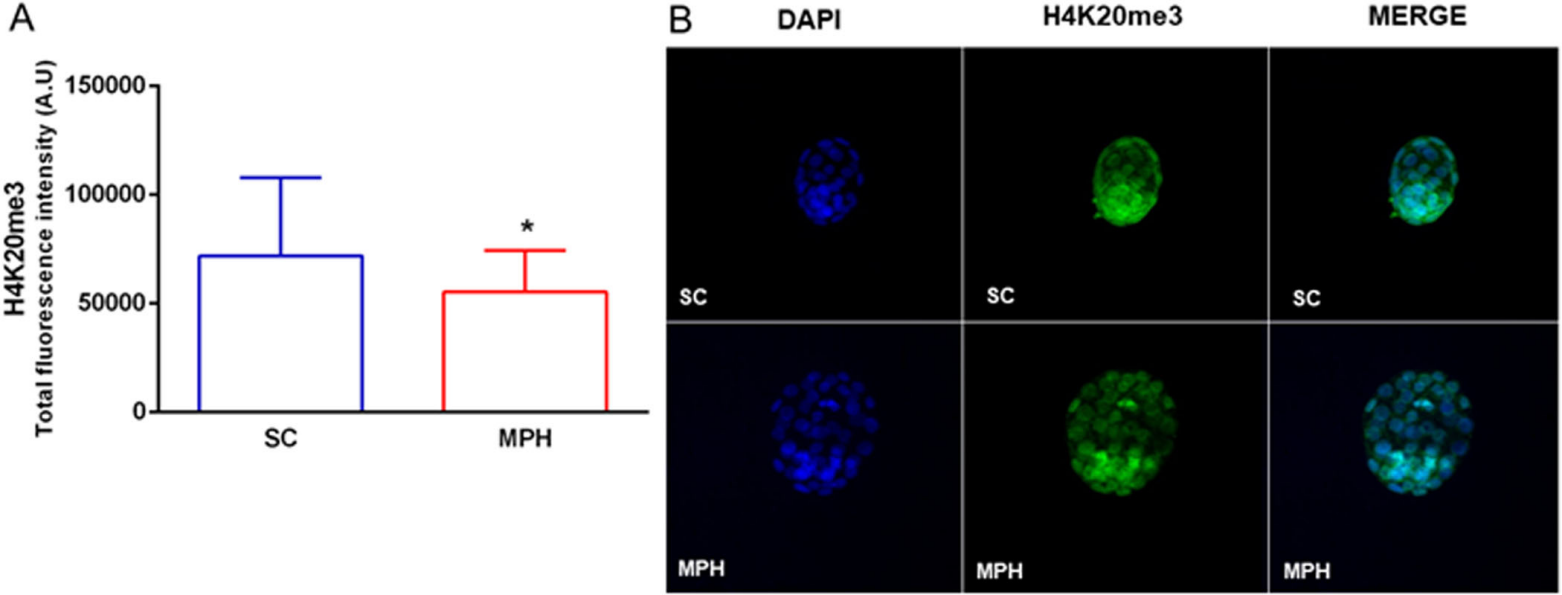
Epigenetic profile of H4K20me3 in blastocysts by immunofluorescence. (A) Blastocysts from the MPH group showed a statistically significant reduction in total fluorescence intensity for H4K20me3 when compared with the SC group (*p = 0.05; *n* of blastocysts/4 males/group, *n* = 25 [SC] and *n* = 24 [MPH]). Statistical analysis was performed using Student's *t* test. (B) Representative image of immunocytochemical staining for H4K20me3 in blastocysts. Nuclei were stained with DAPI (blue) and H4K20me3 with Alexa488 (green). DAPI, 4′,6′‐diamino‐2‐phenyl‐indole; MPH, methylphenidate hydrochloride; SC, sham control.

However, the repressive marks H3K9me3 (Figure 4A,B) and H4K20me3 (Figure 5A,B) showed statistical differences, in opposite ways. While the total fluorescence of blastocysts from the MPH group showed greater intensity for H3K9me3 (*n* = 20 SC; *n* = 32 MPH), it appears reduced for H4K20me3 (*n* = 25 SC; *n* = 24 MPH) when compared with those from group SC.

## 4 Discussion

The ever‐growing number of studies has shown a meaningful relationship between paternal DNA, early embryo development, and future offspring health. Nevertheless, the impacts of drugs such as methylphenidate on paternal inheritance are still poorly explored. The findings taken from our experimental conditions highlight the influence on morphological quality and the establishment of the histone methylation profile of blastocysts because of MPH genotoxicity to mammalian male germ cells. Herein, MPH exposure to paternal DNA disturbed the epigenetic control of the early embryo in its first generation, implicating the modification of epigenetic marks in H3K9me3 and H4k20me3, together with an indicative increase in H3K4me3. These histone marks are all critical signatures for proper embryonic development and seem to be essential for embryo viability (van de Werken et al. 2014; Aoshima et al. 2015; Yoshida et al. 2018; B. González et al. 2020; Lismer et al. 2021).

Conventional sperm analysis is useful for clinical practice in reproductive medicine, presenting a good correlation of sperm motility/morphology and fertilization rates/embryo development, respectively, but not for pregnancy rates (Villani et al. 2022). On the other hand, sperm chromatin integrity has been used as a consistent predictor of preimplantation embryo quality and success rates (Vendramini et al. 2012; Sedó et al. 2017; Borges et al. 2019; Aitken and Lewis 2023; Nguyen et al. 2023). High and low levels of DNA fragmentation cause cleavage stage arrest and other morphokinetics alterations in preimplantation embryos (Ribas‐Maynou et al. 2022; Nguyen et al. 2023), but resilient ones may successfully proceed to the next stage of development (Vendramini et al. 2012; Nguyen et al. 2023). Methods to underpin epigenetic changes in the human embryo are not reliable, which makes it valuable to report a comparison between the blastocyst grading criteria versus key epigenetic marks.

Indeed, subchronic treatment with MPH in prepubertal male rats challenges the nuclear DNA of germ cells by rising sperm chromatin vulnerability, which in our previous report exceeded 20% of DNA fragmentation index in most animals (da Costa Nunes Gomes et al. 2022). The moderate to critically high levels of DNA damage following MPH exposure resulted in almost two times more low‐quality blastocysts, many of those carrying altered epigenetic signatures.

In the protocol used in the current study, the rats had a period of nonexposure to MPH long enough to complete a full spermatogenesis (Vendramini et al. 2012; da Costa Nunes Gomes et al. 2022). According to the germ cell progression suggested by Marchetti et al. (2018), the exposure to a certain toxicant may target germ cells from stem spermatogonia up to pachytene/diplotene spermatocytes. Therefore, MPH seems to affect differentiating spermatogonia—or primary spermatocytes in early stages of the first meiotic division—which remained with persistent DNA damage (da Costa Nunes Gomes et al. 2022) and generated epigenetically disturbed although resilient blastocysts (Vendramini et al. 2012). Our results reinforce the idea that male germ cells during adolescence present a window of vulnerability for epigenetic reprogramming, and that certain aberrations in histone methylation can be delivered to the embryo (Lismer et al. 2021; Barbero et al. 2023).

Sperm epigenome built during spermatogenesis each germ cell stage has specific epimarks that can be dynamically regulated as a response to damage (Gong and Miller 2019; Odroniec et al. 2023). In this matter, under natural conditions within the mouse seminiferous epithelium, a specific increase in H3K4me3 and H3K9me3 is seen during spermatogonial differentiation until early meiosis, which is further decreased in late meiosis. On the other hand, H4K20me3 expression is moderately sustained throughout cellular differentiation (Barbero et al. 2023). These epigenetic signatures promoted by sperm histone PTMs are responsible for regulating the chromatin state (active vs. repressed), and are also essential for DNA repair, since the remodeling of the chromatin structure facilitates this process. Therefore, they have an important role in controlling proper progression of spermatogenesis to deliver the necessary information for the early embryo genome activation, acting as a “stable memory” (Chioccarelli et al. 2020; Barbero et al. 2023).

Since sperm chromatin is not completely epigenetically reprogrammed after fertilization, undue changes in its histone PTMs can be transmitted to the preimplantation embryo (Lismer and Kimmins 2023). As shown in the present study, the morphology of blastocysts fathered by treated males was greatly altered, along with methylation modifications in three paternally inherited histones (Lismer et al. 2021). Corroborating this result, it has already been demonstrated that the cleavage rate and the quality of embryos can be negatively affected due to the increase in the histone to protamine ratio in the sperm chromatin (Fournier et al. 2018). Interestingly, low‐quality blastocysts have been observed in normozoospermic spermatozoa with abnormal methylation profiles of histones (Fournier et al. 2018; Pandya et al. 2024). Although disturbances in the histone to protamine ratio were not significant in sperm exposed to MPH (da Costa Nunes Gomes et al. 2022), the methylation landscape might be affected and should be investigated in more detail to better qualify the origin of the alterations found in the embryos.

As shown in our results, the analysis of histones H3K4me3, H3K9me3, and H4K20me3 using immunocytochemistry revealed changes in the fluorescence intensity patterns in the blastocysts sired from the MPH group, which contained blastomeres more intensely marked in specific areas. However, H3K4me3 epigenetic marks were not statistically significant in the embryos; further analysis to verify these markers on sperm may clarify the relationship of this epigenetic alteration after exposure to MPH, since H3K4me3 increase is associated with genetic activation to double‐strand breaks (DSBs) repair machinery assembly for fixing damaged DNA in spermatocytes (Chen et al. 2020).

Also, it has been shown that the demethylation at the DNA damaged sites is an important step for the damage‐induced chromatin state transition (Gong and Miller 2019; Talibova et al. 2022). Moreover, the spermatogonial phase seems to be especially sensitive to changes in the H3K4me balance linked to epigenetic inheritance (Barbero et al. 2023) and cumulative changes in spermatic H3K4me3 can be associated with a greater disturbance of gene expression in the embryo, as well as an increased severity of developmental anomalies in the offspring (Lismer et al. 2021). Our results for this mark, despite not showing significant differences, suggest a propensity for hypermethylation. One hypothesis to justify this result would be the low number of blastocysts obtained in the MPH group, probably deceased around the morula stage (Liu et al. 2016), caused by the low viability of highly methylated embryos at H3K4.

Alternatively, the histone H3K9me3 is a heterochromatin‐associated repressive mark that is involved in the DNA damage response pathway, playing a critical role in the activation of DSB signaling proteins, such as Tip60 and ataxia telangiectasia mutant. H3K9me3 enrichment must be rapid and temporary, only for this repressive chromatin to inhibit local transcription—by compacting the chromatin structure—limiting DSBs mobility during the early times after DSBs production (Ayrapetov et al. 2014). Besides, it is paternally inherited, carrying a clear functional relevance in the embryo (Wang et al. 2019; Gong and Miller 2019; Lismer and Kimmins 2023). On the other hand, the removal of H3K9me3 is mandatory after fertilization, for creating a less restricted epigenetic environment for the subsequent activation of the zygotic genome and also necessary for the embryo to reach the totipotent state (Wang et al. 2019; Wilson and Krieg 2019; Xu et al. 2021).

Consistent with the literature regarding drugs with similar mechanisms of action as MPH, like, cocaine and methamphetamine, our results demonstrated a greater fluorescence intensity for H3K9me3 in blastocysts from the MPH group. B. González et al. (2020) reported that exposure to cocaine causes changes in the epigenome, increasing H3K9me3 in mouse germ cells. It has been suggested that cocaine, through the activation of the dopamine receptor, could cause an imbalance in this marker, potentially harming embryonic development (B. González et al. 2020). Additionally, Limanaqi et al. (2018) demonstrated that chronic exposure to methamphetamine produces epigenetic effects that further repress gene expression, mainly through increased H3K9 methylation.

Notably, H4K20 methylation is also essential for normal development, contributing to the remodeling and maintenance of embryonic nuclear heterochromatin architecture (Z. Liu et al. 2024). Paternal inherited H4k20me3 has been reported to be propagated through embryonic cleavage divisions (van de Werken et al. 2014), and defects in controlling this epigenetic mark, such as the deletion of its methyltransferase genes, were shown to cause perinatal death in mice (Schotta et al. 2008; Wongtawan et al. 2011). It is worth to mention that there is a relationship between the loss of H4K20me3 and cancer development in humans and animals (Fraga et al. 2005; Pogribny et al. 2006), due to its essential role in the response to DNA damage, especially in the G1 phase of the cell cycle (Svobodová Kovaříková et al. 2018). Therefore, the modifications reported here are concerning elements for the offspring's health.

However, the results involving the presence of the H4K20me3 repressive mark in blastocysts differ a lot across different studies, possibly due to the use of variation in the specificity of the antibodies (Wongtawan et al. 2011; Bonnet‐Garnier et al. 2018; Z. Liu et al. 2024). This mark typically acts in association with H3K9me3 to further compact the chromatin structure. H3k9me3 serves as a docking site for the binding of heterochromatin protein 1 (HP1) isoforms, resulting in less accessible chromatin and, subsequently, HP1 binds to the Suv4‐20h1/2 methyltransferases, then increasing H4K20me3 (van de Werken et al. 2014). Unexpectedly, H4k20me3 was reduced in the blastocysts in the MPH group, under our experimental conditions, possibly as an attempt to activate a compensatory mechanism to maintain embryonic viability facing the increase in H3k9me3.

In summary, paternal exposure to MPH in our experimental conditions triggered epigenetic changes in the blastocyst stage and produced very low‐quality blastocysts. Our results indicate the need for controlled investigations to define if human sperm also carry DNA with defective chromatin following exposure to MPH. In this sense, preconceptional guidance to MPH users during clinical management should be considered, in relation to the possible rise of embryonic loss, or the risk of development of a viable fetus with epigenetic alterations. The implications for the health of the offspring are not yet known and should be investigated soon.

## Author Contributions

**Ana Clara da Costa Nunes Gomes:** investigation, writing – original draft, validation, writing – review and editing, formal analysis, data curation. **Laura Eduarda S. C. Pagliari:** data curation, investigation, methodology. **Taiza Stumpp:** methodology, supervision, writing – review and editing, resources. **Vanessa Vendramini:** conceptualization, investigation, writing – original draft, writing – review and editing, methodology, visualization, validation, formal analysis, project administration, data curation, supervision, resources.

## Conflicts of Interest

The authors declare no conflicts of interest.

## Data Availability

The data that support the findings of this study are available from the corresponding author upon reasonable request.
